# Impact of scatter radiation on spectral quantification performance of first‐ and second‐generation dual‐layer spectral computed tomography

**DOI:** 10.1002/acm2.14383

**Published:** 2024-05-27

**Authors:** Edgar Salazar, Leening P. Liu, Amy E. Perkins, Sandra S. Halliburton, Nadav Shapira, Harold I. Litt, Peter B. Noël

**Affiliations:** ^1^ Department of Radiology University of Pennsylvania Philadelphia Pennsylvania USA; ^2^ Department of Engineering and Architecture Universidad Privada Boliviana La Paz Bolivia; ^3^ Philips Healthcare Orange Village Ohio USA

**Keywords:** Compton scattering artifacts, dual‐layer spectral CT, quantitative CT, spectral computed tomography

## Abstract

**Objective:**

To assess the impact of scatter radiation on quantitative performance of first and second‐generation dual‐layer spectral computed tomography (DLCT) systems.

**Method:**

A phantom with two iodine inserts (1 and 2 mg/mL) configured to intentionally introduce high scattering conditions was scanned with a first‐ and second‐generation DLCT. Collimation widths (maximum of 4 cm for first generation and 8 cm for second generation) and radiation dose levels were varied. To evaluate the performance of both systems, the mean CT numbers of virtual monoenergetic images (MonoEs) at different energies were calculated and compared to expected values. MonoEs at 50  versus 150 keV were plotted to assess material characterization of both DLCTs. Additionally, iodine concentrations were determined, plotted, and compared against expected values. For each experimental scenario, absolute errors were reported.

**Results:**

An experimental setup, including a phantom design, was successfully implemented to simulate high scatter radiation imaging conditions. Both CT scanners illustrated high spectral accuracy for small collimation widths (1 and 2 cm). With increased collimation (4 cm), the second‐generation DLCT outperformed the earlier DLCT system. Further, the spectral performance of the second‐generation DLCT at an 8 cm collimation width was comparable to a 4 cm collimation on the first‐generation DLCT. A comparison of the absolute errors between both systems at lower energy MonoEs illustrates that, for the same acquisition parameters, the second‐generation DLCT generated results with decreased errors. Similarly, the maximum error in iodine quantification was less with second‐generation DLCT (0.45  and 0.33 mg/mL for the first and second‐generation DLCT, respectively).

**Conclusion:**

The implementation of a two‐dimensional anti‐scatter grid in the second‐generation DLCT improves the spectral quantification performance. In the clinical routine, this improvement may enable additional clinical benefits, for example, in lung imaging.

## 1 INTRODUCTION

Spectral computed tomography (CT) is a specialized medical imaging technique that distinguishes itself from conventional CT by enabling the differentiation and analysis of various tissues and materials based on energy‐dependent attenuation. This unique capability provides additional diagnostic information, including effective atomic number maps, iodine density maps, virtual monoenergetic images (MonoEs), and virtual non‐contrast images, among other data.^1^ Consequently, the integration of these advanced imaging features enhances the robustness of diagnostic workflows.^2^, ^3^ The quantitative performance of data derived from spectral CT systems has been demonstrated to be accurate, but performance varies due to external factors such as the scanner type and patient habitus.^4^, ^5^, ^6^ Additionally, Compton‐scattered photons directly affect material decomposition and, subsequently, spectral performance. This can lead to the generation of various artifacts and attenuation shifts that might impact diagnoses where precise quantitative attenuation values or concentrations are required. This scenario is particularly relevant in oncology imaging where increased iodine concentrations are associated with local perfusion within a lesion.^7^, ^8^

Numerous studies have been conducted to evaluate and mitigate the impact of scattered radiation on material decomposition and the resulting spectral outcomes. Both experimental^9^, ^10^, ^11^ and simulated investigations^12^, ^13^, ^14^, ^15^, ^16^, ^17^ have illustrated how intrinsic and extrinsic imaging parameters affect the presence of scattering‐induced artifacts. Techniques aimed at reducing scattering encompass methods such as estimating scattered maps using conventional or artificial intelligence‐based correction algorithms^18^, ^19^, ^20^, ^21^ or incorporating physical instrumentations into the system design to minimize the amount of scattered radiation detected. Examples of these elements include air gaps,^22^ primary modulators,^23^ and anti‐scatter grids.^24^ Among these, anti‐scatter grids stand out as one of the most widely used and accepted solutions in current clinical CT systems. These grids, consisting of lead strips or septa, are strategically placed between the patient and the detector. Their primary function is to absorb, or block scattered radiation while allowing the unscattered, primary radiation to reach the detector, thereby enhancing image quality and diagnostic accuracy. Advanced simulations and experimental studies have been instrumental in fine‐tuning these grids, ensuring they effectively minimize scattering artifacts without compromising the overall image quality or increasing patient radiation dose.

The objective of this study is to perform a quantitative analysis comparing the performance of two dual‐layer spectral CT (DLCT) systems: a first‐generation DLCT (IQon Spectral CT, Philips Healthcare, equipped with a one‐dimensional anti‐scatter grid and has a maximum collimation of 4 cm) and a second‐generation DLCT (Spectral CT7500, Philips Healthcare, equipped with a two‐dimensional anti‐scatter grid and has a maximum collimation of 8 cm). Existing literature^12^, ^13^, ^16^, ^24^ has demonstrated that two‐dimensional grids significantly reduce scattered radiation in comparison to their one‐dimensional counterparts. This reduction in scattered radiation has a potential to increase quantitative performance and diagnostic value.^25^, ^26^ To mimic real‐world scenarios where scattering‐induced challenges can arise, data were collected under conditions simulating sharp transitions between air and water. Such abrupt transitions, commonly encountered in the interfaces between organs like the lung and liver, can potentially compromise diagnostic quality.^27^ Our comparative analysis of the two DLCT systems was conducted based on several parameters. These included evaluating Hounsfield Unit (HU) accuracy across various energy levels of MonoEs, the characterization of materials through spectral attenuation maps, and the measurement of iodine density.

## 2 METHODS

### 2.1 Phantom

**FIGURE 1:**
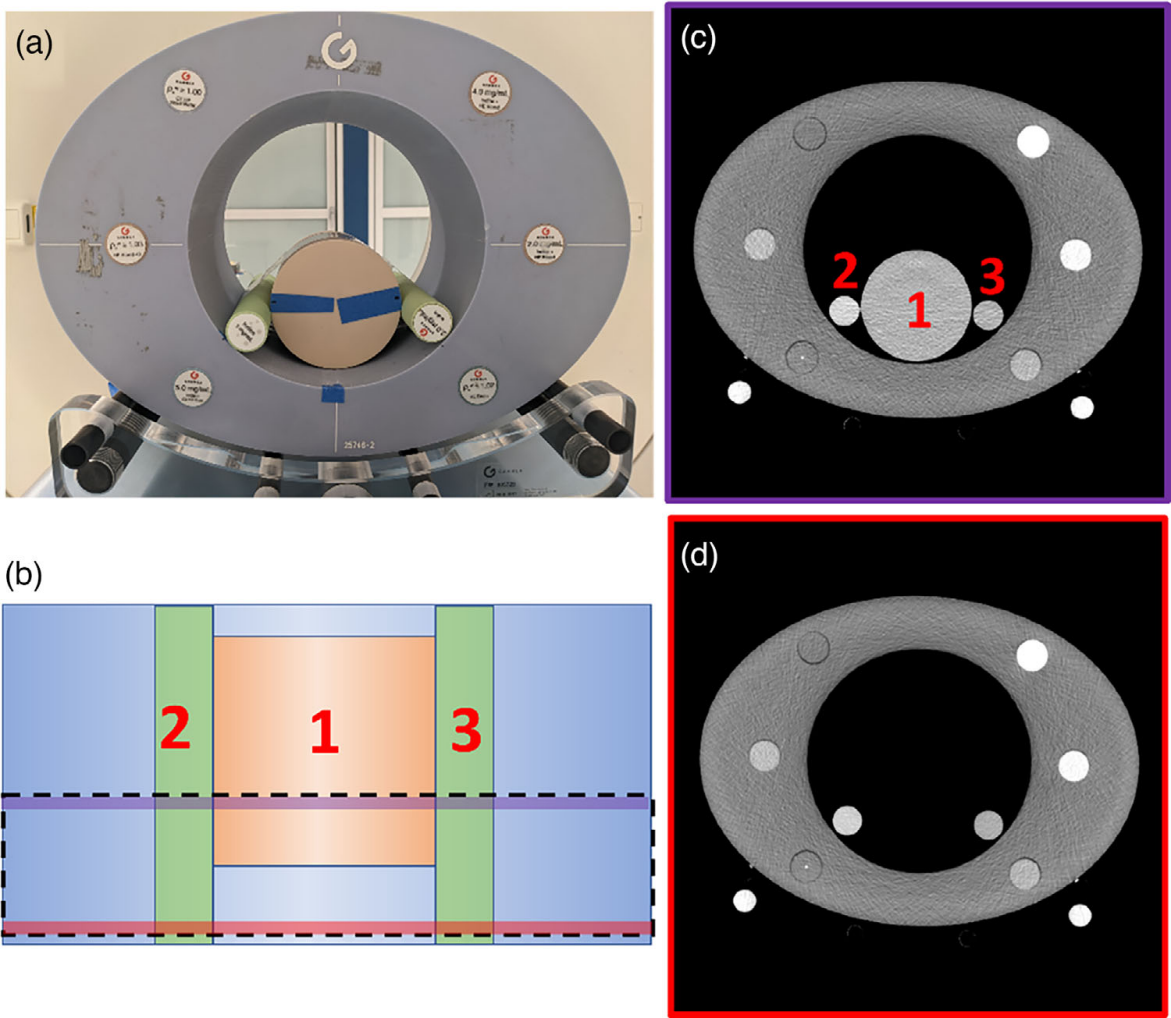
Experimental setup for the evaluation of scatter radiation in first and second‐generation DLCT. (a) Experimental setup and positioning of the phantom. (b) Top view of the phantom's 10 cm water (1), 2 mg/mL iodine (2), and 1 mg/mL iodine inserts (3). (c,d) Cross‐section of the phantom with (c) and without (d) the water‐equivalent phantom insert, on either side of the air‐water insert transition, corresponding to the purple and pink lines in (b).

The implemented phantom (see Figure 1) has several components including (i) an outer 40 cm x 30 cm ring with a 20 cm bore (Multi‐energy CT phantom, Gammex), (ii) a 10 cm water equivalent insert (DM10 QRM, PTW), and (iii) two solid iodine inserts of 1 and 2 mg/mL with 2.6 cm diameter (Gammex). The 10 cm insert as well as the iodine inserts were placed inside the larger phantom.

### 2.2 Image acquisition and reconstruction

The phantom was scanned in axial mode with the first and second‐generation DLCT systems using a tube voltage of 120 kVp and three radiation dose levels, CT dose index ($CTDI_{vol}$) 10, 30, and 50 mGy (default radiation doses for axial scanning are close to 10 mGy for both systems). The axial scanning mode was chosen over the helical mode for better control and reproducibility of the position of the x‐ray source with respect to the phantom. A total of four collimations were utilized: 1, 2, 4, and 8 cm; the largest collimation is only available with the second‐generation DLCT. The scan field of view was set to 45 cm and centered over the water‐air transition region, while the rotation time was set to 0.75 s. For image generation, the iterative reconstruction algorithm (iDose^4^, Philips Healthcare) was set to level 0 (equivalent to conventional FBP),^28^ and a slice thickness of 1 mm was used. A 2 cm scan range of interest was centered in the air‐water transition boundary (see Figure 1). For the 1 cm collimation width, two consecutive scans were performed to cover the 2 cm scan range. Considering all the dose‐collimation combinations, 21 scenarios were evaluated in the experiments, and three independent acquisitions were run per scenario. Derived spectral results included MonoE images at 40, 50, 70, 100, 120, 150, and 200 keV, and iodine density maps. Table 1 summarizes all the acquisition and reconstruction parameters.

**TABLE 1:** Scan acquisition and image reconstruction parameters.

Tube voltage (kVp)	120
CTDI_vol_ (mGy)	10, 30, 50
Collimation (cm)	1, 2, 4, 8 (only for second‐gen. DLCT)
Rotation time (s)	0.75
Field of view (cm)	45
Slice thickness (mm)	1
Image matrix	512 × 512
Pixel spacing (in x and y) (mm)	0.88
Reconstruction filter	B
Reconstruction mode	iDose^4^ level 0

### 2.3 Image analysis

A visual comparison between reconstructed MonoE 40 keV images at all radiation dose levels was performed for each system. In the 20 slices spanning the scan range, a circular region of interest (ROI) was placed within iodine inserts covering 85% of the total cross‐sectional area. This step was performed to avoid potential errors and artifacts from partial volume effects.^29^ The slices outside of the scan range of interest for the 4 cm collimation and the 8 cm collimation (20 and 60 slices, respectively) were not considered in the analysis. Expected attenuations for every MonoE were calculated using the material composition and density information supplied by the manufacturer,^4^ and the following equation:

(1) $$HU\left(E\right)=1000\times \left(\frac{\rho \sum_{i}w_{i}\times \mu_{i}\left(E\right)}{\mu_{water}\left(E\right)}-1\right),$$

where ρ is the physical density, $w_{i}$ is the mass fraction of the element *i*, $\mu_{i}$(E) is the attenuation of the element *i* at energy E, while $\mu_{water}$(E) is the attenuation of the water at energy E. The expected range was calculated using the the physical density tolerance provided by the manufacturer. To assess the performance of the systems, the mean HU for MonoEs ranging from 40  to 200 keV were determined for both iodine inserts and compared to the expected range. The mean absolute HU errors for each MonoE and all dose‐collimation combinations were calculated. To assess the material characterization accuracy, MonoE 50  versus 150 keV data were plotted and compared to expected values.

## 3 RESULTS

### 3.1 Qualitative assessment of scattering artifacts

**FIGURE 2:**
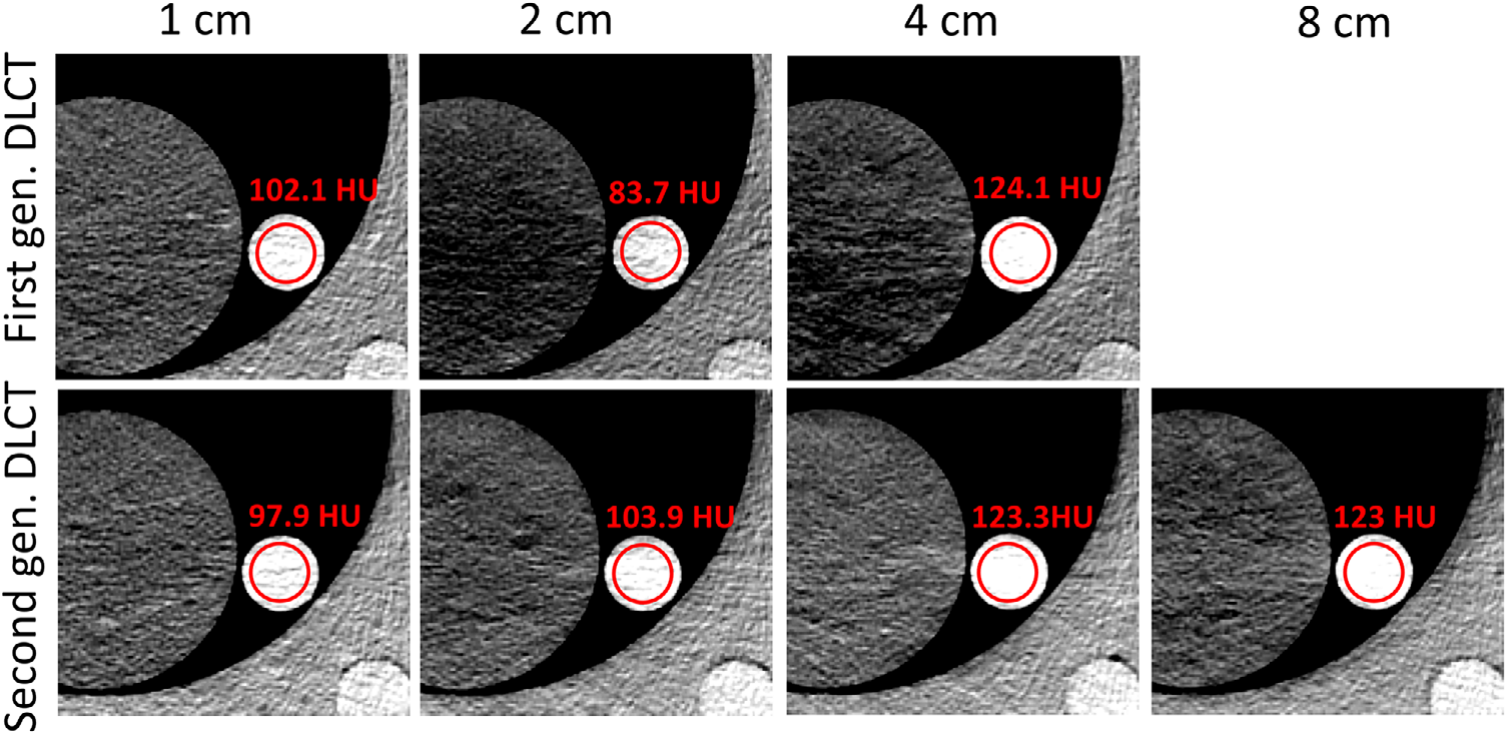
Visual comparison of MonoE 40 keV, 50 mGy image of the 1 mg/mL iodine insert (center) for different collimations scanned with first (top row) and second (bottom row) generation DLCT. W:200, L:0 HU. With increasing collimation, the contrast of the insert changes. At 4 cm collimation, the region of the insert (red circles) with first‐generation DLCT shows more saturated values (white) compared to the second generation DLCT 8 cm collimation. Due to improved scatter correction with the second‐generation DLCT, the surrounding artifacts are significantly reduced.

Figure 2 illustrates the difference between Mono 40 keV images obtained with the first and second‐generation DLCT. It is evident that as the collimation increased, the contrast of both inserts changed due to additional scatter. Upon comparing the first‐generation system at a 4 cm collimation with the second‐generation system at 8 cm collimation, the enhanced performance of the second‐generation DLCT became qualitatively and quantitatively discernible.

### 3.2 Mean attenuation for different MonoEs

**FIGURE 3:**
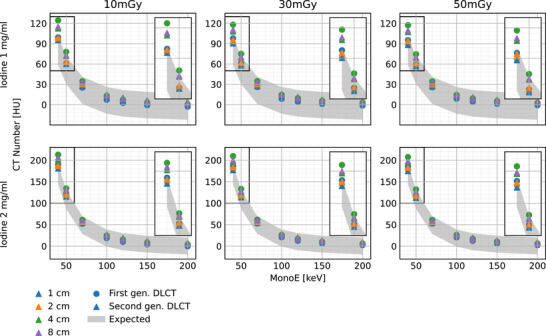
CT numbers for MonoE images ranging from 40 to 200 keV for the two iodine inserts (1  and 2 mg/mL). Deviation from the expected range can be observed at lower MonoE values (A maximum deviation of 45.7 HU for 40 keV, and 27.2 HU for 50 keV). No significant deviation from the nominal values is observed at middle to high MonoE values.

Figure 3 shows the mean CT number evolution over MonoEs ranging from 40 to 200 keV for the different collimations and radiation doses, and for both inserts, where the shaded light‐gray area represents the expected range. In all the scenarios, except for lower MonoEs (40 and 50 keV), the mean HU fell within the expected range. However, when comparing the absolute errors between both systems at lower energies, it is evident that, under the same acquisition parameters, the second‐generation DLCT yielded results closer to expected values (up to 45.7 HU for the first‐generation DLCT and 39.2 HU for the second‐generation DLCT, both at 40 keV). For both systems, increasing the radiation dose for a fixed collimation reduced the error. Moreover, reducing the collimation width for a fixed radiation dose decreased the absolute error in most cases.

The mean of the standard deviations of the attenuation similarly illustrates a dependency on the collimation width at low energies. For the first‐generation DLCT MonoE 40 keV and 50 mGy, a change from 1 to 4 cm in collimation increased the standard deviation in 3.2 and 2.9 HU for the 1 and 2 mg/mL iodine inserts, respectively. For the second‐generation DLCT MonoE 40 keV and 50 mGy, a change from 1 to 8 cm in collimation increased the standard deviation in 5.6 and 4.5 HU, for the 1 and 2 mg/mL inserts, respectively. With a fixed collimation, increasing the radiation dose decreased the mean standard deviation at all energies. With the first‐generation DLCT, MonoE 40 keV, and 4 cm collimation width, increasing the radiation dose from 10 to 50 mGy decreased the mean standard deviation in 2.2 and 4.7 HU for the 1 and 2 mg/mL inserts, respectively. The same change in dose for the second‐generation DLCT yielded a decrease of 1.6 and 1.8 HU for the 1 and 2 mg/mL inserts, respectively, at 8 cm collimation.

### 3.3 Material characterization

**FIGURE 4:**
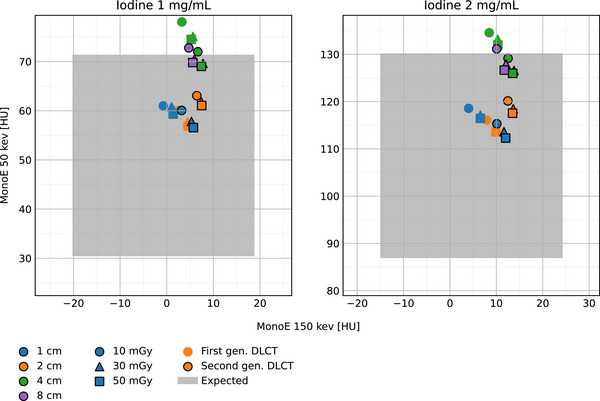
MonoE 50 keV versus MonoE 150 keV for 1 mg/mL and 2 mg/mL iodine inserts. At 4 cm collimation, all data points fall outside the expected range for the first‐generation DLCT. For the second‐generation DLCT, only one case (Iodine 1 mg/mL, 10 mGy) falls outside the expected range when using 4 cm collimation and two cases (Iodine 1  and 2 mg/mL, both at 10 mGy) fall outside when using 8 cm collimation.

Figure 4 shows the MonoE 50  versus 150 keV plot of attenuation for both inserts. The expected range is represented by the shaded light‐gray area. For both systems, all mean CT numbers fell within the expected range when using 1 or 2 cm collimation widths. When the collimation is increased to 4 cm, one case (10 mGy, 1 mg/mL iodine insert) fell outside the range for the second‐generation DLCT (0.6 HU above the expected MonoE 50 keV upper limit), while all of the first‐generation DLCT cases fell outside the range with the largest deviation (6.7 HU above the expected MonoE 50 keV upper limit) also measured at 10 mGy for the 1 mg/mL iodine insert. When the collimation is increased to 8 cm (only available for second‐generation DLCT), the CT numbers at 10 mGy fell outside the range for both inserts (1.4 and 0.8 HU above the expected MonoE ‐50 keV upper limit for the iodine 1 and 2 mg/mL inserts, respectively).

### 3.4 Iodine density quantification

**FIGURE 5:**
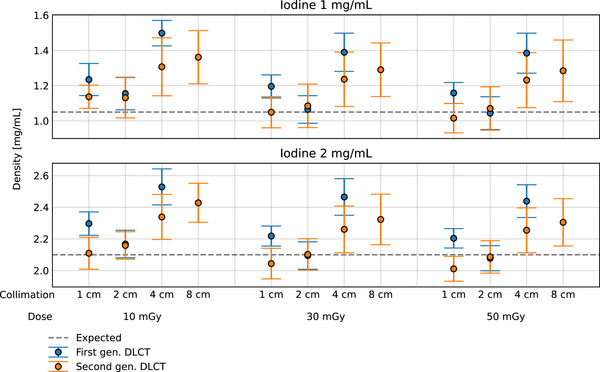
Iodine density quantification for the 1  and 2 mg/mL iodine inserts. The error bars represent the mean standard deviation of the pixels inside the ROIs. For both systems and both inserts, the widest collimation produces an increased deviation from the expected range.

Figure 5 shows the experimental iodine density quantification for the 1 and 2 mg/mL iodine inserts, respectively; the blue markers represent the first‐generation DLCT, while the orange markers represent the second‐generation DLCT. A comparison between the two systems for the same acquisition parameters showed that iodine quantification values for the second‐generation DLCT were closer to expected values. At 4 cm collimation and 50 mGy dose, the absolute error for the first‐generation DLCT was 0.33 and 0.34 mg/mL for the 1 and 2 mg/mL iodine inserts, respectively, while for the same collimation and dose, errors for the second‐generation DLCT were 0.18 and 0.15 mg/mL, respectively. For both systems, decreasing the collimation from 4 to 1 cm for a fixed radiation dose decreased the error. For the first‐generation DLCT, at 10 mGy dose, for example, the absolute error decreased from 0.45 mg/mL at 4 cm to 0.18 mg/mL at 1 cm for the 1 mg/mL iodine insert. For the second‐generation DLCT, a decrease from 0.26 to 0.09 mg/mL was observed at 10 mGy with the 1 mg/mL iodine insert. A similar behavior was observed for the 2 mg/mL iodine insert when collimation was decreased from 4 to 1 cm. Interestingly, for first‐generation DLCT quantification was best at 2 cm, not 1 cm; at a dose of 10 mGy, the absolute error at 2 cm was 0.10 mg/mL for the 1 mg/mL insert and 0.20 mg/mL for the 2 mg/mL iodine insert. A direct comparison between the first‐generation DLCT at 4 cm collimation and the second‐generation DLCT at 8 cm collimation showed that the absolute error was lower for the latter across all dose values. At a dose of 50 mGy and collimation of 4 cm, for example, absolute error for the first‐generation DLCT was 0.33 mg/mL, while for the same dose and collimation of 8 cm, absolute error was 0.24 mg/mL.

The mean standard deviation of the iodine densities for both systems showed a direct dependency on the collimation width for a fixed radiation dose. For the first‐generation DLCT, at a fixed 50 mGy dose, the standard deviation decreased from 0.11 to 0.06 mg/mL and from 0.10 to 0.06 mg/mL when the collimation was reduced from 4 to 1 cm for the 1 and 2 mg/mL iodine inserts, respectively. For second‐generation DLCT, the standard deviation decreased from 0.17 to 0.08 mg/mL and from 0.15 to 0.08 mg/mL when the collimation was reduced from 8 to 1 cm for the 1 and 2 mg/mL iodine inserts, respectively (refer to Table 2 for specific quantitative results).

**TABLE 2:** Absolute error and mean standard deviation in iodine density (mg/mL) measured in both iodine inserts from iodine density images.

			Absolute error (mg/mL)		Standard deviation (mg/mL)	
System	CTDI_vol_ (mGy)	Collimation (cm)	Iodine 1 (mg/mL) insert	Iodine 2 (mg/mL) insert	Iodine 1 (mg/mL) insert	Iodine 2 (mg/mL) insert
First‐gen. DLCT	10	1	0.18	0.20	0.09	0.07
		2	0.10	0.07	0.09	0.09
		4	0.45	0.43	0.07	0.11
	30	1	0.15	0.12	0.07	0.06
		2	0.02	0.00	0.08	0.09
		4	0.34	0.36	0.11	0.12
	50	1	0.11	0.10	0.06	0.06
		2	0.00	0.02	0.09	0.08
		4	0.33	0.34	0.11	0.10
Second‐gen. DLCT	10	1	0.09	0.01	0.07	0.10
		2	0.08	0.06	0.11	0.09
		4	0.26	0.24	0.16	0.14
		8	0.31	0.33	0.15	0.12
	30	1	0.00	0.06	0.09	0.10
		2	0.03	0.00	0.12	0.10
		4	0.19	0.16	0.15	0.15
		8	0.24	0.22	0.15	0.16
	50	1	0.03	0.09	0.08	0.08
		2	0.02	0.01	0.12	0.10
		4	0.18	0.15	0.16	0.14
		8	0.23	0.20	0.17	0.15

*Note*: The expected iodine concentration was taken as the ground truth.

## 4 DISCUSSION

In this study, we introduced and utilized a phantom capable of generating scatter to evaluate the performance of first and second‐generation DLCT systems with varying collimation widths and radiation doses. A comparison of systems was based on attenuation measured from a range of MonoEs and iodine density measured from iodine maps, using expected values as the ground truth. Results show the robustness of both systems to scattering artifacts, with deviations only for certain acquisition parameters. When compared to recent quantification studies,^4^, ^6^, ^25^, ^26^ the observed numerical inaccuracies are more evident, in particular for iodine densities, where absolute errors for DLCT systems do not exceed 0.17 mg/mL at low scatter conditions as proved by Sellerer et al.,^30^ while in our evaluation at high scatter conditions the absolute error reaches 0.45 mg/mL. This confirms the effectiveness of the proposed phantom for determining the effect of scatter on spectral quantification accuracy.

The MonoE attenuation curves for both DLCT systems illustrate that for higher energies (70, 100, 120, 150, 200 keV), the measured data are within the nominal range even with high scatter. For lower energies (40, 50 keV), deviations from the expected range are observed, while smaller collimations (1 and 2 cm) and higher radiation doses (50 mGy) offer better attenuation accuracy. Iodine density values demonstrated a similar behavior, with smaller collimations (1 and 2 cm) as the best scenario for quantification accuracy. At the same time, the dependecy on radiation dose is not as signficant as for MonoE attenuation. In terms of material characterization (MonoE 50  vs. 150 keV), most of the scenarios fall inside the expected region for both DLCT systems; however, for larger collimations the second‐generation DLCT outperforms the first generation.

When both systems are compared at identical radiation doses and a collimation width of 4 cm, the second‐generation DLCT shows quantitative results closer to the expected values and ranges for iodine densities and MonoE attenuation. Further, the second‐generation DLCT at 8 cm collimation offers increased accuracy compared to the first‐generation DLCT at 4 cm collimation. This improvement in the performance is partly due to the two‐dimensional anti‐scatter grid that was incorporated in the second‐generation DLCT. Meyer et al. showed similar results for improved pediatric imaging with the second‐generation DLCT.^25^

The present study, however, had some limitations in its experimental design that could have affected data acquisition. The overall phantom configuration was kept relatively simple but considered sufficient to assess the performance of DLCT systems under high‐scattering conditions. Although the air‐water transition in this basic phantom was instrumental in introducing scattering artifacts to the acquired data, it was limited in its ability to replicate the complexity of actual clinical scenarios. To address this, future experiments will incorporate dedicated 3D‐printed phantoms designed to closely resemble human lung anatomy,^31^ alongside ex vivo samples, to provide a more accurate representation. Additionally, due to the lack of complete symmetry in this phantom, manual positioning of the ROIs was necessary for data analysis, which might have introduced some inaccuracies in the results.

## 5 CONCLUSION

The quantitative performance of both first and second‐generation DLCT systems was assessed under high‐scattering conditions. The introduction of techniques aimed at mitigating scatter radiation effects significantly enhances spectral performance, particularly when employing wider collimation widths. In routine clinical practice, the enhanced performance of second‐generation DLCT systems holds the potential to further enhance the applicability of spectral CT for various diagnostic tasks.

## AUTHOR CONTRIBUTIONS

Peter B. Noël and Nadav Shapira conceived the presented idea. Edgar Salazar contributed to the experimental data acquisition, data compiling, analysis of the results, and to the writing of the manuscript. Leening P. Liu and Nadav Shapira contributed to the experimental data acquisition, analysis of the results, and the writing of the manuscript. Amy E. Perkins, Sandra S. Halliburton, Peter B. Noël, and Harold I. Litt. contributed to the analysis of the results, and to the writing of the manuscript.

## CONFLICT OF INTEREST STATEMENT

The authors declare no conflicts of interest.
